# The impact of perioperative fluid therapy on the short-term outcomes after laparoscopic colorectal cancer surgery with ERAS protocol: a prospective observational study

**DOI:** 10.1038/s41598-023-49704-y

**Published:** 2023-12-14

**Authors:** Magdalena Pisarska-Adamczyk, Grzegorz Torbicz, Natalia Gajewska, Piotr Małczak, Piotr Major, Michał Pędziwiatr, Michał Wysocki

**Affiliations:** 1 Department of Medical Education, Jagiellonian University Medical College, Kraków, Poland; 2 Department of General Surgery and Surgical Oncology, Ludwik Rydygier Memorial Hospital, Kraków, Poland; 3 2nd Department of General Surgery, Jagiellonian University Medical College, Jakubowskiego 2, 30-688 Kraków, Poland

**Keywords:** Cancer, Gastrointestinal cancer, Colorectal cancer

## Abstract

The main goals of the Enhanced recovery after surgery (ERAS) protocol are focused on shortening the length of hospital stay (LOS), expediting convalescence, and reducing morbidity. A balanced perioperative fluid therapy is among the significant interventions incorporated by the ERAS protocol. The article contains extensive discussion surrounding the impact of this individual intervention on short-term outcomes. The aim of this study was to assess the impact of perioperative fluid therapy on short-term outcomes in patients after laparoscopic colorectal cancer surgery. The analysis included consecutive patients, who had undergone laparoscopic colorectal cancer operations between 2013 and 2020. Patients were divided into two groups: restricted (≤ 2500 ml) or excessive (> 2500 ml) perioperative fluid therapy. A standardized ERAS protocol was implemented in all patients. The study outcomes included recovery parameters and the morbidity rate, LOS and 30 days readmission rate. There were 361 and 80 patients in groups 1 and 2, respectively. There were no statistically significant differences between the groups in terms of demographic parameters and factors related to the surgical procedure. Logistic regression showed that restricted fluid therapy as a single intervention was associated with improvement in tolerance of diet on 1st postoperative day (OR 2.18, 95% CI 1.31–3.62, *p* = 0.003), accelerated mobilization on 1st postoperative day (OR 2.43, 95% CI 1.29–4.61, *p* = 0.006), lower risk of postoperative morbidity (OR 0.58, 95%CI 0.36–0.98, *p* = 0.046), shorter LOS (OR 0.49, 95% CI 0.29–0.81, *p* = 0.005) and reduced readmission rate (OR 0.48, 95% CI 0.23–0.98, *p* = 0.045). A balanced perioperative fluid therapy on the day of surgery may be associated with faster convalescence, lower morbidity rate, shorter LOS and lower 30 days readmission rate.

## Introduction

Multi-element ERAS protocol was proven to accelerate convalescence, reduce perioperative morbidity and shorten the length of hospital stay after colorectal surgery^1,2^. Previous studies have shown that increase in compliance (from high to very high/complete compliance) has also been associated with improvement in short-term outcomes^3^. Until now it is not known which elements of the protocol play a key role in this improvement. A balanced perioperative fluid therapy seems to be one of the most significant elements. Recently published articles included speculations that excessive perioperative fluid therapy may have been increasing the complication rate^4,5^.

The aim of this study was to assess the impact of perioperative fluid therapy on the short-term outcomes after laparoscopic colorectal cancer surgery combined with Enhanced Recovery After Surgery protocol.

## Methods

### Inclusion and exclusion criteria

Adult patients with histologically confirmed adenocarcinoma of colon or rectum, who had undergone elective laparoscopic surgeries between 2013 and 2020 were included in the observational study prospectively, and gathered in the study database. Exclusion criteria were: emergency or planned open surgery, multivisceral resection, transanal endoscopic microsurgery (TEM), concomitant inflammatory bowel diseases, need for conversion or intensive care unit stay immediately after surgery.

**Figure 1:**
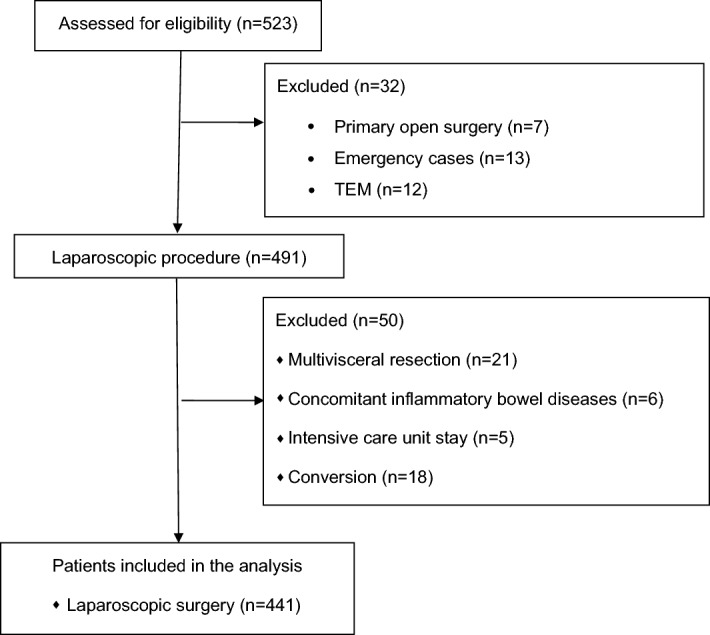
Patients flow through the study.

Figure 1 shows patients flow through the study.

Study data, such as compliance with the ERAS protocol, demographics, cancer stage, location of primary lesion, intraoperative parameters, short-term outcomes (including perioperative morbidity, mortality, length of hospital stay (LOS), readmission rate), were collected in the prospective database.

For the purposes of further analysis, the entire group was divided into two groups, depending on the amount of intravenous fluids administered on the day of surgery: ≤ 2500 ml (Group 1) and > 2500 ml (Group 2). The cut-off value was established based on the literature and previous ERAS recommendations, considering that these are laparoscopic patients who are euvolemic at the time of surgery^6^. It has been shown that IV fluid loads above 3000 mL for colonic resections and 3500 mL for rectal resections during the day of surgery increased morbidity^7^. These data, however, refer to open procedures, so the cut-off points in our case are modified.

Groups were compared in terms of demographic factors and short-term outcomes.

### Perioperative fluid management

The study analyzed both the total amount of IV fluids on the day of surgery, as well as the IV fluid intake per kilogram of the patient’s body weight. The amount of fluids administered was counted from the morning hours (7 am) preceding the surgery for the next 24 h. After returning from the operating room, the patient could start drinking fluids orally if no postoperative nausea or vomiting were present. According to our protocol we do not continue infusions postoperatively when not needed. Perioperative fluid therapy included crystalloids, colloids and blood transfusion. During the operation and 4 h after, the decision on perioperative fluid therapy was made by the anesthesiologist, and after the patient was returned to the ward, the fluids were administered by the colorectal surgeon.

In all patients prophylaxis of postoperative nausea and vomiting (PONV) was implemented: Dexamethasone 8 mg IV, Ondansetron 8 mg IV, Metoclopramide 10 mg IV, in various combinations depending on the risk.

### Outcomes

The primary outcome was perioperative morbidity rate. The secondary outcomes were recovery parameters (tolerating oral diet and mobilization on the first postoperative day), length of hospital stay and 30 days readmission rate.

Perioperative morbidity was graded according to the Clavien-Dindo classification.

In all patients the 16-item ERAS protocol was applied. Its principles were based on the ERAS Society Guidelines^6–8^.

### Statistical analysis

Data were analyzed using Statsoft STATISTICA v.13 (StatSoft Inc., Tulsa, OK, USA). A descriptive study was conducted on the sample. Numerical variables are presented as mean ± standard deviation (SD) and as median with interquartile range (IQR) when appropriate. Categorical variables are presented as frequencies and percentages. The odds ratio (OR) with 95% confidence intervals (CI) was used to present the results of logistic regression. To examine the relationship between each variable and the outcome, the Pearson chi-square test of independence was used. The Shapiro–Wilk test was conducted to assess the normal distribution of data. Normally distributed quantitative data were analyzed with the Student's t-test. In the case of skewed quantitative variables, the Mann–Whitney U test was applied. Finally the logistic regression models were analyzed. A *p* value < 0.05 was considered statistically significant.

### Ethical approval

The study was approved by the Jagiellonian University Ethics Review Committee (approval number 1072.6120.225.2017). All procedures have been performed in accordance with the ethical standards laid down in the 1964 Declaration of Helsinki and its later amendments.

Informed consent for proposed surgical treatment was obtained from all patients before surgery.

## Results

During the study period, a total of 523 patients with colorectal cancer were operated on in our unit. 32 patients did not meet inclusion criteria. Out of the remaining 491 patients, 21 had multivisceral resection, 18 were converted, 5 needed ICU stay immediately after surgery, and 6 patients had concomitant inflammatory bowel disease. Overall, 441 patients underwent laparoscopic resections of colorectal cancer and were included in the study group (Fig. 1).

There were no statistically significant differences between groups in terms of demographic parameters, such as: gender, age, body mass index, ASA scale, presence of comorbidities, localization and stage of the tumor. The demographic analysis of the groups is shown in Table 1.

**Table 1:** Demographic analysis of patient groups.

Parameter	Group 1 ≤ 2500 ml	Group 2 > 2500 ml	*p* value
Number of patients, n	361	80	–
Females, n (%)	167 (46.3%)	33 (41.3%)	0.415
Males, n (%)	194 (53.7%)	47 (58.7%)	
Mean age, years ± SD	64.5 ± 13.6	64.2 ± 13.3	0.869
BMI, kg/m^2^ ± SD	26.3 ± 4.9	26.7 ± 4.2	0.354
ASA 1, n (%)	12 (3.3%)	3 (3.8%)	0.988
ASA 2, n (%)	224 (62.1%)	48 (60.0%)	
ASA 3, n (%)	116 (32.1%)	27 (33.8%)	
ASA 4, n (%)	9 (2.5%)	2 (2.5%)	
Any comorbidity, n (%)	252 (69.8%)	55 (68.8%)	0.853
Cardiovascular, n (%)	115 (31.9%)	30 (37.5%)	0.331
Hypertension, n (%)	194 (53.7%)	36 (45.0%)	0.157
Diabetes, n (%)	73 (20.2%)	10 (12.5%)	0.11
Pulmonary disease, n (%)	36 (10.0%)	6 (7.5%)	0.496
Renal disease, n (%)	29 (8.0%)	4 (5.0%)	0.485
Liver disease, n (%)	13 (3.6%)	3 (3.8%)	0.949
AJCC Stage I, n (%)	98 (27.2%)	20 (25%)	0.768
AJCC Stage II, n (%)	132 (36.6%)	27 (33.8%)	
AJCC Stage III, n (%)	91 (25.2%)	21 (26.3%)	
AJCC Stage IV, n (%)	40 (11.1%)	12 (15.0%)	
Right hemicolectomy, n (%)	128 (35.5%)	24 (30.0%)	0.288
Left hemicolectomy, n (%)	16 (4.4%)	5 (6.25%)	
Sigmoid resection, n (%)	72 (19.9%)	18 (22.5%)	
Colectomy, n (%)	8 (2.2%)	5 (6.25%)	
Rectum resection, n (%)	137 (38.0%)	28 (35.0%)	
Formation of stoma, n (%)	96 (27.0%)	16 (21.3%)	0.306
Mean operative time, min. ± SD	193.4 ± 62.9	211.4 ± 76.3	0.099
Mean intraoperative blood loss, ml ± SD	116.6 ± 116.5	140.1 ± 149.5	0.604
Years 2013–2016	176	44	0.312
Years 2017–2020	185	36	
Mean compliance with ERAS protocol, % ± SD	82.1 ± 14.7	79.6 ± 15.2	0.212

Mean compliance with the ERAS protocol was comparable between Group 1 and Group 2 (*p* = 0.222). There was also no statistically significant difference between the groups in the Pre-operative carbohydrate loading 79.9% versus 72.5% (*p* = 0.209).

The median perioperative volume of IV fluids (24 h) in the entire group was 2000 ml (IQR 1500–2500 ml) and 27.8 ml (IQR 20.6–36.5 ml) per 1 kg of body weight. We did not observe a statistically significant difference in the percentage of patients requiring IV fluid on the 1st postoperative day. Data on perioperative fluid therapy are presented in Table 2.

**Table 2:** Perioperative fluid management in analyzed groups.

Parameter	Group 1 ≤ 2500 ml	Group 2 > 2500 ml	*p* value
Median total IV 24 h fluid, ml (IQR)	2000 (1500–2000)	3000 (3000–3500)	< 0.05
Median IV 24 h fluid administration per 1 kg body weight, ml (IQR)	25.32 (19.05–31.25)	43.75 (37.74–48.70)	< 0.05
Median last IV fluid administration, day (IQR)	1 (0–3)	2 (1–3)	0.006
1^st^ POD, n (%)	74 (20.5%)	20 (25.0%)	0.374

*Complications* The overall morbidity rate was 31%. There was a significant difference in morbidity rate between Group 1 and Group 2 (27.4% vs. 38.8%, *p* = 0.044). There were no statistical differences in their severity according to Clavien-Dindo classification (*p* = 0.634).

Recovery parameters: Tolerance of full oral diet on the first postoperative day (76% vs. 59%, *p* = 0.022) and mobilization on the day of surgery (90% vs. 78%, *p* = 0.005) were significantly better in Group 1. There were no differences between groups in the use of opioids (*p* = 0.6395) and in time to the first flatus (*p* = 0.102).

The median LOS in Group 1 was 4 days, which differed from Group 2, where it was 5 days (*p* = 0.0007). Hospital readmission occurred in 28 (7.8%) patients in Group 1 and in 12 (15%) patients in Group 2 (*p* = 0.041). The postoperative outcomes are presented in Table 3.

**Table 3:** Postoperative outcomes in analyzed groups.

Parameter	Group 1 ≤ 2500 ml	Group 2 > 2500 ml	*p* value
Number of patients, n	361	80	–
Tolerating oral diet on the first postoperative day, n (%)	273 (76%)	47 (59%)	0.002
Mobilization on the first postoperative day, n (%)	325 (90%)	63 (78%)	0.005
No postoperative use of opioids, n (%)	194 (54.8%)	41 (51.9%)	0.64
Time to first flatus, days ± SD	1.96 ± 1.91	2.14 ± 1.40	0.102
Patients without complications, n (%)	262 (72.6%)	49 (61.3%)	0.044
Patients with complications, n (%)	99 (27.4%)	31 (38.8%)	
Clavien-Dindo 1, n (%)	40 (11%)	15 (18.8%)	0.634
Clavien-Dindo 2, n (%)	23 (6.4%)	6 (7.5%)	
Clavien-Dindo 3, n (%)	25 (6.9%)	9 (11.3%)	
Clavien-Dindo 4, n (%)	6 (1.7%)	1 (1.3%)	
Clavien-Dindo 5, n (%)	5 (1.4%)	–	
Clinical relevant anastomotic leakage (CD 3–5), n (%)	28 (7.75%)	8 (10%)	0.507
Mean length of hospital stay, days ± SD	6.00 ± 6.98	8.09 ± 9.22	< 0.05
Median length of hospital stay, days (IQR)	4 (3–6)	5 (4–7)	
Readmission, n (%)	28 (7.8%)	12 (15.0%)	0.041

Univariate logistic regression models showed that restricted fluid therapy was increasing odds for improved tolerance of diet on 1st postoperative day (OR 2.18, 95% CI 1.31–3.62, *p* = 0.003), accelerated mobilization on 1st postoperative day (OR 2.43, 95% CI 1.29–4.61, *p* = 0.006), while reduced odds ratio of perioperative morbidity (OR 0.58, 95% CI 0.36–0.98, *p* = 0.046), shortened length of hospital stay (OR 0.49, 95% CI 0.29–0.81, *p* = 0.005), and reduced readmission rate (OR 0.48, 95% CI 0.23–0.98, *p* = 0.045).

## Discussion

Results from this study showed that balanced perioperative fluid therapy was associated with better convalescence, lower morbidity rate, shorter length of hospital stay and lower 30 days readmission rate in patients who had undergone laparoscopic colorectal cancer operations. These results were observed in groups with comparable overall compliance with the ERAS protocol.

Fundamental issue in this research was to define a balanced and excessive perioperative fluid therapy. Significant differences were found in the literature between authors in setting the cut-off point. Moreover, they used different names ‘standard’, ‘overload’, ‘liberal’, ‘restricted’, ‘balanced’ regarding perioperative IV fluid therapy^9,10^. Asklid et al.^11^ divided patients into two groups—with less than 3000 ml and with > 3000 ml on the day of surgery including crystalloids, colloids and blood transfusions. Authors of the cited meta-analysis divided all patients from 9 included articles into 3 groups: restricted (< 1.75 L/day), balanced (1.75–2.75 L/day) and liberal/overload fluid therapy (> 2.75 L/day)^9^. A recently published study set the cut-off for excessive perioperative IV fluid for the open colorectal surgery to 3500 ml^12^. In our study, the chosen cut-off point was 2500 ml. This value was lower than the stated above, but applied only to patients operated with laparoscopic access. There is no gold standard for setting the cut-off point of excessive perioperative fluid therapy, so the numbers and definitions differ between studies and inevitably are the source of bias in meta-analysis.

Although it is generally known that the ERAS protocol improves short-term results, little is known about which particular elements are playing a key role. So far we have no confirmation in the form of scientific evidence, but perioperative fluid therapy poses as one of the key elements.

Some data suggest that fluid overload in the perioperative period may increase the morbidity rate, including one of the most severe—the anastomotic leakage^13–15^. Fluid overload may lead to intestinal edema, inhibit gastrointestinal transit, and impair anastomotic healing. These data appear to be uncertain due to the high heterogeneity of studies, different perioperative care protocols, and various definitions of the balanced fluid therapy. In some of the works, only the perioperative period is analyzed, while in others, the longer time is counted, e.g. 72 h. Pache et al.^12^ indicated, that it was not an excessive perioperative fluid administration (> 3.5 L), but in their opinion, the ERAS compliance of less than 70% and weight gain more than 3.5 kg at second postoperative day were the independent risk factors for overall complication.

In our study, balanced fluid administration on the day of surgery was associated with a lower overall complication rate. However, it did not affect their severity according to Clavien-Dindo classification. In our study group, there was an association with the improved tolerance of diet and accelerated mobilization on the first postoperative day. Both of these were related to the shorter length of hospital stay. However, its impact on readmission seemed surprising. Analyzing the reasons for hospital readmission, it seemed that it had resulted from transient nausea and vomiting, which occurred less frequently in the group with balanced perioperative fluid therapy.

It seemed that the solution would be the use of goal-directed intravenous fluid therapy to improve clinical outcomes, based on some clinical investigations. It was confirmed by a meta-analysis, which showed that goal-directed IV fluid therapy significantly decreased mortality and surgical morbidity in moderate and high-risk surgical patients^16^. Its use in each patient was associated with difficulties due to the invasiveness and advancement of the techniques used. Its Implementation is challenging in a common clinical practice. Fortunately, there have also been studies showing that in some groups of patients it was not necessary. As for the patients undergoing minimally invasive techniques, in which perioperative care is based on modern multimodal programs. This was confirmed by Gómez-Izquierdo et al. in a randomized-controlled trial^17^. They showed that the intraoperative goal-directed fluid therapy, compared with a fluid therapy based on traditional principles, did not reduce primary postoperative ileus in the patients undergoing laparoscopic colorectal surgery in a context of the ERAS protocol. Also, other recovery measurements of gastrointestinal function were similar. Hence, it seems that goal-directed fluid therapy may not be necessary in a group of patients undergoing laparoscopic surgery combined with the ERAS protocol.

Although perioperative fluid therapy is not a new topic, it seems to be a key element influencing patient outcomes. The issue was raised by recently published studies that describe its potential impact on long-term results^18,19^. Asklid et al.^11^ reported a possible association between restrictive IV fluid management on the day of surgery and colorectal cancer-specific 5 years survival. For that reason more research is needed to increase the understanding of the relationship of the perioperative fluid therapy and long-term oncological outcomes.

This study has several limitations that are typical of a single-center study. This study was a prospective observational study potentially biased with confounding by indication. We only analyzed the short-term results (< 30 days after surgery). We also analyzed together patients with colon and rectal cancer, which also may have created bias. The compliance of the ERAS protocol in our group was not equal during the whole study period and was the lowest at the early stage. Additionally, the perioperative fluid therapy was also influenced by other factors, such as: age, comorbidities, extent of surgery, which affects the time of surgery and intraoperative blood loss.

## Conclusions

A balanced perioperative fluid therapy on the day of surgery as a single intervention may change the short-term results after laparoscopic colorectal cancer resections. It may be associated with a faster convalescence, lower morbidity rate, shorter LOS and lower 30 days readmission rate.

### Supplementary Information

Supplementary Information.

## Data Availability

The data that support the findings of this study are not openly available due to reasons of sensitivity and are available from the corresponding author upon reasonable request.

## References

[1] Greco M, Capretti G, Beretta L, Gemma M, Pecorelli N, Braga M (2014). Enhanced recovery program in colorectal surgery: A meta-analysis of randomized controlled trials. World J. Surg..

[2] Group, ERAS Compliance (2015). The impact of enhanced recovery protocol compliance on elective colorectal cancer resection: Results from an international registry. Ann. Surg..

[3] Pisarska M, Pędziwiatr M, Małczak P, Major P, Ochenduszko S, Zub-Pokrowiecka A (2016). Do we really need the full compliance with ERAS protocol in laparoscopic colorectal surgery? A prospective cohort study. Int. J. Surg..

[4] Simpson RG, Quayle J, Stylianides N, Carlson G, Soop M (2017). Intravenous fluid and electrolyte administration in elective gastrointestinal surgery: Mechanisms of excessive therapy. Ann. R. Coll. Surg. Engl..

[5] Rollins KE, Lobo DN (2016). Intraoperative goal-directed fluid therapy in elective major abdominal surgery: A meta-analysis of randomized controlled trials. Ann. Surg..

[6] Gustafsson UO, Scott MJ, Hubner M, Nygren J, Demartines N, Francis N (2019). Guidelines for perioperative care in elective colorectal surgery: Enhanced recovery after surgery (ERAS(®)) society recommendations: 2018. World J. Surg..

[7] Nygren J, Thacker J, Carli F, Fearon KC, Norderval S, Lobo DN (2012). Guidelines for perioperative care in elective rectal/pelvic surgery: Enhanced recovery after surgery (ERAS®) Society recommendations. Clin. Nutr..

[8] Pędziwiatr M, Pisarska M, Major P, Grochowska A, Matłok M, Przęczek K (2016). Laparoscopic colorectal cancer surgery combined with enhanced recovery after surgery protocol (ERAS) reduces the negative impact of sarcopenia on short-term outcomes. Eur. J. Surg. Oncol..

[9] Varadhan KK, Lobo DN (2010). A meta-analysis of randomised controlled trials of intravenous fluid therapy in major elective open abdominal surgery: Getting the balance right. Proc. Nutr. Soc..

[10] Gustafsson UO, Ljungqvist O (2011). Perioperative nutritional management in digestive tract surgery. Curr. Opin. Clin. Nutr. Metab. Care.

[11] Asklid D, Segelman J, Gedda C, Hjern F, Pekkari K, Gustafsson UO (2017). The impact of perioperative fluid therapy on short-term outcomes and 5-year survival among patients undergoing colorectal cancer surgery - A prospective cohort study within an ERAS protocol. Eur. J. Surg. Oncol..

[12] Pache B, Hübner M, Solà J, Hahnloser D, Demartines N, Grass F (2019). Receiver operating characteristic analysis to determine optimal fluid management during open colorectal surgery. Colorectal Dis..

[13] Lobo DN, Bostock KA, Neal KR, Perkins AC, Rowlands BJ, Allison SP (2002). Effect of salt and water balance on recovery of gastrointestinal function after elective colonic resection: A randomised controlled trial. Lancet.

[14] Schnüriger B, Inaba K, Wu T, Eberle BM, Belzberg H, Demetriades D (2011). Crystalloids after primary colon resection and anastomosis at initial trauma laparotomy: Excessive volumes are associated with anastomotic leakage. J. Trauma.

[15] Brandstrup B, Tønnesen H, Beier-Holgersen R, Hjortsø E, Ørding H, Lindorff-Larsen K (2003). Effects of intravenous fluid restriction on postoperative complications: Comparison of two perioperative fluid regimens: A randomized assessor-blinded multicenter trial. Ann. Surg..

[16] Hamilton MA, Cecconi M, Rhodes A (2011). A systematic review and meta-analysis on the use of preemptive hemodynamic intervention to improve postoperative outcomes in moderate and high-risk surgical patients. Anesth. Analg..

[17] Gómez-Izquierdo JC, Trainito A, Mirzakandov D, Stein BL, Liberman S, Charlebois P (2017). Goal-directed fluid therapy does not reduce primary postoperative ileus after elective laparoscopic colorectal surgery: A randomized controlled trial. Anesthesiology.

[18] Pisarska M, Torbicz G, Gajewska N, Rubinkiewicz M, Wierdak M, Major P (2019). Compliance with the ERAS protocol and 3-year survival after laparoscopic surgery for non-metastatic colorectal cancer. World J. Surg..

[19] Rubinkiewicz M, Pisarska M, Zarzycki P, Truszkiewicz K, Witowski J, Su M (2020). High compliance to ERAS protocol does not improve overall survival in patients treated for resectable advanced gastric cancer. Wideochir Inne Tech Maloinwazyjne.

