# Supplementary material for: The impact of perioperative fluid therapy on the short-term outcomes after laparoscopic colorectal cancer surgery with ERAS protocol: a prospective observational study
Source: Sci Rep. 2023 Dec 14;13:22282. doi: 10.1038/s41598-023-49704-y (PMC10721599; doi:10.1038/s41598-023-49704-y)
Supplement: Supplementary file 1 — Supplementary Information. [file 41598_2023_49704_MOESM1_ESM.pdf]

**ERAS protocol used in our department (2013-2020)**
1. Preoperative counseling and patient`s education
2. No bowel preparation (oral lavage in case of low rectal resection with TME and defunctioning loop ileostomy)
3. Pre-operative carbohydrate loading (400 ml of Nutricia preOp® 2 hours prior surgery)
4. Antithrombotic prophylaxis (Clexane® 40 mg sc. starting in the evening prior surgery)
5. Antibiotic prophylaxis (preoperative Cefuroxime 1,5 g + Metronidazole 0,5 g iv 30-60 min. prior surgery)
6. Laparoscopic surgery
7. Balanced intravenous fluid therapy (<2500 ml intravenous fluids during the day of surgery, less than 150 mmol sodium)
8. No nasogastric tubes postoperatively
9. No drains left routinely for colonic resections, one drain placed for <24 h in case of TME
10. TAP block and standard anesthesia protocol
11. Avoiding opioids, multimodal analgesia (oral when possible - Paracetamol 4x1g, Ibuprofen 2x200 mg, Metamizole 2x500 mg, or Ketoprofen 2x100 mg)
12. Prevention of postoperative nausea and vomiting (PONV) (Dexamethasone 8 mg iv., Ondansetron 8 mg iv., Metoclopramide 10 mg iv.)
13. Postoperative oxygenation therapy (4-6 l/min.)
14. Early oral feeding (oral nutritional supplement 4h postoperatively - Nutrcia Nutridrink® or Nestlé Impact®, light hospital diet and oral nutritional supplements on the first postoperative day, full hospital diet in the second postoperative day)
15. Urinary catheter removal on the first postoperative day
16. Full mobilization on the first postoperative day (getting out of bed, going to toilette, walking along the corridor, at least 4 hours out of bed)
